# Surgeons’ knowledge regarding perioperative pain management in patients with opioid use disorder: a survey among 260 members of the American College of Surgeons

**DOI:** 10.1186/s13037-024-00392-1

**Published:** 2024-03-04

**Authors:** Jessica R. Burgess, Kathleen C. Heneghan, Tarra G. Barot, Jonah J. Stulberg

**Affiliations:** 1 Department of Surgery, Eastern Virginia Medical School, 6th Floor Hofheimer Hall, 825 Fairfax Avenue, Norfolk, VA 23507 USA; 2 Division of Education, American College of Surgeons, Chicago, IL USA; 3 Department of Surgery, University of Texas Houston, Houston, TX USA

**Keywords:** Opioid use disorder, Postoperative analgesia, Opioid dependence, Perioperative management

## Abstract

**Background:**

Patients with opioid use disorder (OUD) are increasing, challenging surgeons to adjust post-operative pain management guidelines. A literature review identified limited information on how to best care for these patients. The purpose of this study was to determine surgical perioperative management of OUD, challenges, and support needed for optimal care.

**Methods:**

This study utilized an anonymous voluntary survey that was distributed to members of the American College of Surgeons through the association’s electronic weekly newsletter. The survey was advertised weekly for three consecutive weeks. The survey included questions regarding surgeons’ management of perioperative pain in patients with opioid use disorder and perceived barriers in treatment.

**Results:**

A total of 260 surgeons responded representing all specialties except ophthalmology. General surgery (66.5%) and plastic and reconstructive surgery (7.5%) represented the majority of responders. Ninety-five percent of surgeons reported treating a patient who used opioids in the past month and 86% encountered a patient with OUD. Nearly half (46%) reported being uncomfortable managing postoperative pain in patients with OUD. Most (67%) were not aware of any guidelines or standards pertaining to perioperative management of patients with OUD. While consultation was sought by 86% of surgeons, analyses identified lack of timely response and a lack of care coordination among specialists. Lack of knowledge and fear of harm (contributing further to addiction) were the most common themes.

**Conclusion:**

Nearly half of surgeons report discomfort caring for patients with OUD with the vast majority involving a consulting service to assist with their care. Most surgeons believe that it would be helpful to have guidelines regarding the care of these patients. This provides an opportunity for increased education and training on the perioperative management of patients with OUD and further collaboration with addiction medicine, psychiatry and pain management colleagues.

## Introduction

As the opioid epidemic continues in the United States, surgeons and their professional organizations have led efforts to reduce the amount of opioids prescribed and to prevent opioid use disorder [1–4]. The use of screening tools has been helpful in identifying patients currently using opioids, or patients at a higher risk for continued use or overdose [5]. Studies have identified that 25% of patients are already using opioids at the time of surgery [6]. The ACS opioid survey reported a similar finding with surgeons identifying that 25% of patients used opioids in the month prior to surgery [7]. This is a significant challenge to surgical professionals with a recent pooled analysis from 33 studies of over 1.9 million patients identifying that the risk factors associated with prolonged opioid use following surgery included preoperative use of opioids or illicit cocaine and a preoperative diagnosis of back pain [8].

A recent study estimated that 7% of patients develop persistent chronic opioid use after undergoing surgery, with a fraction of these patients developing opioid use disorder (OUD) [9]. OUD is a psychiatric diagnosis defined by the DSM-V as the chronic use of opioids that causes clinically significant distress or impairment [10] As the second highest prescribers of opioids, surgeons have been at the forefront of the opioid epidemic and working towards prevention in the postoperative population [11].

Despite the heightened awareness and educational focus on OUD prevention, there has been a lack of education and training for surgeons on the perioperative treatment of patients with OUD. Patients with OUD undergoing surgery have increased length of stay, increased pain and a higher complication rate than their counterparts [12–15]. While these studies have shown worse outcomes for OUD patients, standardized guidelines for the surgical community on how to better care for these patients other than preventative strategies are limited. In a 2019 review by the VA “Evidence Brief: Managing Acute Pain in Patients with Opioid Use Disorder on Medication -assisted Treatment” it was identified that the guidelines that are available are often based on case studies and expert consensus, with no clear steps for clinicians to take in acute pain scenarios [16].

A survey of surgeons and trainees in 2020 by Ayakta and colleagues identified that a majority of respondents had not received any training regarding prescribing opioids and pain control [17]. Patients with OUD have been shown to experience increased pain and poorer pain control postoperatively which makes education regarding appropriate perioperative management even more necessary in this population [14, 15]. In addition to lack of education and training on this topic, there is a significant amount of negative stigma when caring for these patients. A survey study of cardiac surgeons by Tiako and colleagues identified that access to addiction medicine services is limited and that a quarter of surgeons did not consider OUD a disease or felt that medication assisted therapy (MAT), such a buprenorphine or methadone, reduced recurrence [18].

The authors hypothesized that surgeons do not feel comfortable treating perioperative pain in patients with OUD. The objectives of this study were to determine whether surgeons felt comfortable treating this patient population and to identify the perceived barriers to appropriately treating these patients.

## Methods

### Survey design

A survey was developed to determine surgeons’ opinions regarding perioperative pain management in patients with opioid use disorder. Demographic data included years in practice and specialty. Quantitative questions included the percent of patients seen using opioids in the past month, number of patients with OUD seen in the last month, comfort treating patients with OUD, use of prescription drug monitoring programs, availability of guidelines, and if the surgeon utilizes other specialties in the care of these patients. Qualitative questions included five single, open ended questions. The qualitative questions were developed by the American College of Surgeons Safe Pain Control workgroup. Qualitative questions included:

- What are the most common clinical scenarios in which your specialty encounters patients with OUD?
- What are the most common challenges faced by your specialty when working with surgical patients with OUD?
- What is your specialty’s approach to harm reduction?
- What are the barriers for using medication (such as buprenorphine, methadone and naltrexone) for OUD in the hospital?
- What can hospital psychiatrists do to support your specialty?

### Data collection

The survey was administered via email as a part of the American College of Surgeons weekly newsletter, the ACS Bulletin. This online newsletter was sent to the membership of the American College of Surgeons which includes approximately 80,000 members. Surveys with incomplete responses were excluded. Surgeons were recruited to participate via the newsletter weekly over three consecutive weeks in November of 2020. All specialties were invited to participate. Data collection was completed using REDCap© (Research Electronic Data Capture) which is a secure, web-based application. The survey was considered IRB exempt. The introduction to the survey informed participants of the purpose of the study and that no identifiable data would be collected. Participants acknowledged review of this and agreed to complete the survey. A record ID was generated and no identifiable data was collected.

### Data analysis

Descriptive statistics and analysis of frequency data were used to assess categorical variables. A Kendall’s tau-b correlation was run to determine strength and association between variables.

Qualitative analysis was performed on the five single open-ended questions. Each qualitative response was considered and independent unit of analysis, with content coding and domains determined separately. This was done by two separate reviewers within the research team with an initial list of 15 domains. Each participant’s comments were then classified into the appropriate domain. Any comments that did not have agreement between the two separate reviewers were discussed between reviewers and consensus obtained. Following a review of the domains with comments, the 2–3 main domains for each question were identified. The data was recoded based on the central themes to calculate the results of the analysis using frequencies and percentages.

## Results

Two hundred and sixty surgeons participated in the study. The mean years in practice is 19.5 years (range 0–50 plus years) with the highest percentage of surgeons in practice less than 10 years (32%). The majority of surgeons represented General Surgery (66.5%), followed by Plastic and Reconstructive Surgery (7.5%) and all other specialties represented less than 5 % each of the total sample.

Surgeons reported a large range of patients who were using opioids at the time of their surgical consult ranging from 0 to 95% percent of the patient population they treated, with a mean of 15.4% or nearly 1 of 6 using opioids in the month prior to surgery. The majority (30.7%) reporting a range of 10–19% of patients using opioids in the past month and only 4% of surgeons reported no patients using opioids in the month prior to surgery. Surgeons’ encounters with patients using opioids are described in Table 1.

**Table 1:** Surgeon report of patient opioid use, comfort and use of guidelines and, PDMP check

Patient Opioid Use in the Month Prior to Surgery		
Range 0–95%	Mean 15.4%	
0	12 (4.5%)	
1–9	66 (24.9%)	
10–19	80 (30.7%)	
20–29	41 (15.8%)	
30 +	32 (12.7%)	
Unknown/no idea	28*(10.8%)	
Patients with OUD in the Past Month		
Range 0–75	Mean 4.92	
None	33 (12.7%)	
1–9	127 (60.5%)	
10–19	28 (10.8%)	
20 plus	16 (6.2%)	
Unknown	26 (10%)	
Guidelines Available For Managing OUD		
Yes	74 (28.5%)	
No	174 (66.5%)	
Comfort Managing OUD		
Yes	141 (45.8%)	
No	112 (54.2%)	
Surgeons Checking the PDMP		
Practice Years	PDMP Yes	PDMP No
0–10	54 (65.8%)	28 (32.2%)
11–20	36 (66.6%)	18 (33.3%)
21–30	47 (87.2%)	29 (38.2%)
31–50+	41 (87.2%)	6 (12.8%)
Total	170 (65.6%)	80 (30.8%)

The number of patients with OUD was encountered less frequently. Table 1 shows an average of 5 patients having OUD with a range of 0 to 75 patients per month. Most surgeons reported encountering less than 5 patients with OUD in the month prior to surgery (66%) with the majority from that group (40%) reporting one per month.

The Prescription Drug Monitoring Program (PDMP) was checked by 68% (170/259) of surgeons. In a review of age groups and how often they check the PDMP, surgeons in practice over 31 years, checked the PDMP most often (41/47, 87%) vs. 6/87, 12.8% not checking). The mean years in practice for those stating yes for checking was 20.7 years and mean practice years for those stating no was 17.5 years.

The majority (174/260, 67%) did not have guidelines or standards available on managing perioperative pain for patients with OUD. For the 28% that did have guidelines, 60% (45/74) identified the guidelines were developed by their practice site/hospital and 38% identified the guidelines/standards were available from their specialty association. Utilizing a crosstabs summary as seen in Table 2, a higher proportion (73%) of those utilizing guidelines (54/74) identified confidence in caring for patients with OUD than those not having guidelines and expressing confidence (20/74). For those with who did not identify using any endorsed guidelines, the proportion of those with no guidelines and comfort was equal to those with no guidelines and discomfort level in managing OUD. When asked about the helpfulness of having guidelines by the ACS, the majority of those responding to the question identified that yes it would be helpful (60%), but nearly one-third (35%) did not respond to this question.

**Table 2:** Guideline availability and comfort managing OUD patients

	Comfort Managing OUD		Total
	No	Yes	
Guidelines Available			
No	86	87	174
Yes	20	54	74
Total	112	141	260

A Kendall’s tau-b correlation was run to determine the relationship between guideline availability and comfort. There was a strong, positive correlation between comfort and guideline availability which was statistically significant (τb = 5.226, *p* = .000). There was no significant correlation between surgeon comfort with management and the volume of patients treated monthly with current opioid use or OUD

Surgeons were asked four questions regarding managing patients with OUD (See Tables 3 and 4). A total of 208–228 of the 260 participants responded to the open ended questions. In response to challenges faced when working with patients with OUD, there were two broad categories: a lack of knowledge and a lack of resources. Lack of knowledge was cited by 66% of surgeons. This included how to adjust medication and dosing, a lack of training in prescribing higher than usual amounts of opioids for postoperative pain control, and an overall lack of experience in managing pain in patients with OUD. Another area under lack of knowledge was communication and expectation setting when confronted with requests for medication refills and normal postoperative expectations. Under lack of resources, surgeons identified the need for specialists to work with them to manage patients with OUD. This also included patient education resources to guide patients on how to appropriately utilize analgesics postoperatively (i.e., for short-term, acute surgical pain control and not for other conditions such as insomnia).

**Table 3:** Surgeons’ challenges and barriers when working with patients with OUD

Questions: What are the most common challenges faced by your specialty when working with surgical patients with OUD? (*n* = 228) What are the barriers for using medication (such as buprenorphine, methadone, naltrexone) for OUD in the hospital? (*n* = 217)		
Theme	Subtheme	Quote
Lack of Knowledge (65%)	Surgeons are not trained nor educated to prescribe MAT	Need training on additional pain medication to treat acute postoperative pain on top of chronic dose. Poor understanding of how to prescribe (dosing) and/or manage these medications or their side effects combined with a lack of physicians to then follow those patients.
	Lack of experience	I don’t have experience managing substance abuse for patients who need acute pain control. The patient’s pain specialist is reluctant to prescribe medications acutely after surgery, despite doses in excess of my comfort level and training.
	Fear of contributing further to patient’s addiction	It is difficult balancing the control of postoperative pain with pain seeking behavior (diversion).
	Communication and patient expectation setting	Most patients have low postoperative pain scores but use opioids for sleep, feeling relaxed, and manage pain at other non-operative sites. Patients don’t see the harm in taking the medication provided to them, approaching it in a naïve way.
Lack of Resources (29%)	Lack of access to (pain/addiction) specialists/lack of care coordination	Sometimes it’s hard getting in touch with the methadone clinic to work out missed doses and formulate a plan of care. Buprenorphine has largely been regulated to pain management and palliative care but pain management does not prescribe for outpatients.
	Lack of infrastructure (EHR); restrictive regulations by state/insurance	(Institutional hurdles) Existing standard order sets include easily ordered high opioid dosing. Hard to decrease dosing or not order for those patients stating no opioids. If surgeon order high opioid doses, they could be subject to discipline. On the other hand, they could be cited for poor patient pain control.
	Lack of transition of care following postoperative pain control to maintenance therapy and dosing	There is a lack of coordination of care with their pain management/addiction physician. Insurance doesn’t cover the cost always of non-opioid alternatives. Unfortunately, it’s easier to prescribe an opioid.

Table 3 synthesizes the challenges when working with patients with OUD. The barriers to include two main themes: a lack of knowledge (cited by 65%) and a lack of resources (cited by 29%). Surgeons cite a lack of guidelines, a lack of training in prescribing higher than usual amounts of opioids for postoperative pain control, and an overall lack of experience in managing pain in patients with OUD. Additionally, standard order sets make it easy for surgeons to over-prescribe opioids to patients who may not necessarily need all of them. Surgeons cited a lack of training when prescribing medication assisted treatment (MAT) and the need for guidance to balance postoperative pain control while not worsening a patient’s OUD. The need for specialists to work with surgeons to manage OUD and the need for patient education to teach how to use analgesics correctly for acute post-operative pain versus other issues are also needed

Surgeons’ approach to harm reduction and perspectives on psychiatrist involvement in the treatment of patients with OUD

**Table 4:** Surgeons’ approach to harm reduction and perspective on psychiatrist involvement in treatment of patient with OUD

Question: What is your specialty’s approach to harm reduction (ie, helping the patient to use less, engage in less risky behaviors [providing only 5–7 days of an opioid prescription, care coordination, prescribing 20–50 MME daily, co-prescribing naloxone])? (*n* = 225)		
Theme	Subtheme	Quote
Medication management (75%)	Limiting dose or duration (46%)	Give only a short prescriptions - 3 days for acute pain. Give only a fixed amount – usually 7–10 days I don’t know my specialty’s approach, but I will order a finite number of pills with no refills – firm.
	Use Alternatives - multimodal pain management (23%)	I use alternatives included: acetaminophen, gabapentin, Robaxin®, and sometimes other alternative medications.
	Use Narcan/naloxone (6%)	Co-prescribe naloxone, maximize non-opioids, minimize number of opioid pills, short-term follow up with outpatient pain / addiction specialist Prescribe minimal amount of pills, prescribe naloxone and involve a family member if there is a trustworthy one
Patient Education 23%	Expectation setting (10%)	Limit prescribing: Max of 20 tablets, less commonly 30. Expectation setting from initial visit to be off of any additional medications by the end of the prescription along with the expectation that there will not be refills after the immediate post-op period, usually just the single post-op prescription.
	Specialist referral (13%)	Support patients with another specialist to educate and counsel on addiction management – pain specialist, addiction medicine, nursing, as appropriate.
Use Published Guidelines (2%)		We are monitoring postoperative opioid prescribing very closely and aiming to meet the Michigan OPEN guidelines by prescribing only a 3 days supply and only prescribe naloxone if prescribing > 50 MME/day.
Question: What can hospital psychiatrists do to support your specialty (what would their ideal role be in terms of team treatment or collaboration)? (*n* = 230)		
Be available and engage throughout the process (64%)	Have a presence in the hospital and help in the perioperative setting.	We don’t have any psychiatrists available however their assessment and review of polypharmacy would be helpful. Quick availability to assess and co-manage patients with OUD in the perioperative setting. This list could go on for a long time. I think they should round on almost all trauma and complex EGS patients. We do not have a psychiatrist in house or to consult, only through telemedicine, and there’s no follow up after discharge, and they don’t consult for pain management either.
	Counselling and coordination by other specialists	Psychiatrists should be helping to provide patient counselling Psychiatrist and addiction counselors are needed to support expectation setting, clarify roles and assist with care coordination for the post operative period. Help treat conditions that exacerbate the pain experience, such as depression and anxiety and direct care away from medications to behavioral and distraction techniques
	Be involved in the process	Institute automatic referrals to psychiatry when a patient has OUD. Provide psychiatric evaluation and consult to ensure patient is using appropriate coping mechanisms; help with treatment plan, ensure post-discharge follow up by psychiatry.
Professional Education (13%)	Protocols/guidelines/review polypharmacy	Psychiatrists are comfortable with medications such as buprenorphine but are often not comfortable with the administration of narcotics / acute pain management.
	Staff Education and how to outreach for consultation	Education of staff, students, residents and colleagues, plus patients on OUD management in the perioperative setting. Identify their role as consults – how can we utilize them to optimize patient recovery – patient expectation setting, treatment of co-morbidities, care coordination post-discharge, medication management.
Involve Pain Specialists (15%)	Pain and Addiction Specialists	Care coordination with addiction medicine as appropriate with referral to pain specialist. I wonder if a pain specialist would be better than a psychiatrist to help treat/collaborate with patients chronic pain/addiction issues.
Uncertain of how psychiatry could help (8%)		Psychiatrists are not available in the hospital; I don’t know how they can help.

Harm reduction involved two main themes which included medication management (cited by 64%) and patient education (cited by 23%). The majority of surgeons reported limiting the number of prescription opioid pills and/or refills as well as employing multimodal pain management including regional anesthesia and over the counter analgesics. A minority of respondents also reported co-prescribing naloxone when the patient’s opioid prescription dose was > 50 MME, in accordance with CDC guidance. Similarly, surgeons are aware of the Michigan OPEN prescribing guidelines for opioid prescriptions following various surgeries and are moving towards adopting them at their own institutions. Regarding how psychiatry can help, the majority of comments revolved around the lack of psychiatrist availability (cited by 64%) as well as their lack of follow-up with patients, post-discharge and the failure of the hospital system to ensure care coordination through the lack of formal multidisciplinary care teams

Surgeons’ responses to barriers to medication management for OUD was reported by 208 participants. The main categories included lack of knowledge (37%) a lack of resources (35%) and no barriers or didn’t know of any (28%). Surgeons cited a lack of training when prescribing medication assisted treatment in the hospital and the need for guidance to help them balance postoperative pain control while still managing and not worsening a patient’s OUD. The lack of resources included both lack of access to the pain/addiction teams or methadone clinics and lack of support for the transition of care. Resources also included the infrastructure issues such as restrictions with state, legal prescribing and standardized order sets.

With respect to harm reduction with OUD patients, the majority of surgeons reported limiting the number of prescription opioid pills and/or refills (46%) and multimodal pain control (23%). Multimodal pain management including regional anesthesia and over the counter analgesics. Specialist referral was cited by 13% along with patient education (10%). Naltrexone administration (6%) and guideline use (2%) were rarely identified. Co-prescribing naloxone when the patient’s opioid prescription dose was > 50 MME was one quote which was in accordance with CDC guidance. Most surgeons (85%) identified utilizing physicians of other specialists to assist in the management of perioperative pain for a patient with OUD.

When surgeons were asked about the most common clinical scenarios where they encountered patients using opioids or with OUD, the majority stated pre-operatively due to chronic pain, not related to surgery, cancer related pain and for trauma patients who continued to use opioids and needed additions operations. Post-operatively was less common, but surgeons encountered patients reporting opioid use after not responding to pain medications and demonstrating signs of withdrawal.

## Discussion

This survey of 260 surgeons across multiple specialties highlights the surgeon perceived challenges regarding the management of patients with opioid use disorder as well as the barriers to their care. OUD is an increasingly prevalent condition in America and this survey found that the percentage of surgical patients with OUD is highly variable. Almost half of surgeons reported that patients with OUD comprise less than 5% of their patients, however, 13% of surgeons reported that up to a third of their patients have OUD. Specialties that had the highest reported proportions of patients with OUD were trauma, general surgery, vascular and orthopedics/hand. A national survey in 2015 found that while over a third of adults used prescription opioids, 16.7% of those reported an OUD; this equates out to 0.8% of the adult population or 1.9 million people [19]. Over 80% of surgeons reported caring for at least one patient per month with OUD. A 2015 survey of hand surgeons also highlighted the prevalence of opioid use disorder with 76% of respondents believing that opioid use was a large problem in their communities [20].

This survey also highlights surgeons’ use of their states’ respective Prescription Drug Monitoring Programs (PDMP). Thirty-seven states have a PDMP with 16 states mandating that it be utilized by prescribers. Studies have shown conflicting results regarding the effect that PDMP utilization has on opioid prescribing habits however, most show an overall decline in the amount of opioids prescribed in states with mandatory PMPs [21–23]. This survey of surgeons found that 62% of surgeons regularly use their state’s PDMP. Interestingly, there was a trend towards increased PMP utilization among surgeons with more years in practice. In surgeons with less than 10 years in practice, 65.8% reported using a PDMP, in contrast to 87% of surgeons with over 30 years in practice. It is unclear the reasons behind this trend and may simply be a reflection of increased experience in opioid prescribing and interest in using available resources.

While many medical and surgical organizations have best practice guidelines on opioid prescribing and preventing chronic opioid use, very few surgical organizations have published guidelines regarding the care of patient with OUD. There was recently a consensus statement from 15 professional surgical and medical organizations with guiding principles for the perioperative management of patients with chronic opioid use or OUD [24]. The Centers for Disease Control also has standardized guidelines for the use of prescription opioids in patients with chronic opioid use which are referenced by the American Urological Association. Several states, including Michigan and Pennsylvania have comprehensive online resources and best practice guidelines on the topic. While there are multiple general guidelines published by medical and psychiatric organizations, surgeons would benefit from surgery specific guidelines to help address preoperative and postoperative pain control in this patient population. Only one third of surgeons had guidelines available, and the majority of these were practice/hospital based (44/75–59%) and only (28/75) 37% were developed by the specialty association. Almost 90% of those answering the question, agreed that it would be helpful to have published guidelines available online. Almost all (96%) of surgeons who reported feeling uncomfortable caring for patients with OUD felt that having published guidelines from the American College of Surgeons would be helpful. This highlights the need that surgeons have for increased education and training in caring for this patient population.

Almost half of respondents reported being uncomfortable managing patients with OUD. When asked what the most common challenges were, two thirds reported challenges that were related to a lack of knowledge. This included a lack of familiarity with commonly used medication assisted treatment (MAT) such as methadone and buprenorphine, lack of experience or training and concern that they will inadvertently contribute to their OUD or abuse of medication. Twenty nine percent of surgeons cited a lack of resources including poor access to consultants and institutional hurdles. A common concern was the inability to effectively transfer the patient’s care to an addiction specialist or pain management specialist once their acute surgical issues have resolved. In 2019, the American Association of Medical Colleges (AAMC) reported that there are only 3500 practicing addiction specialist physicians in the country to treat the 20.7 million Americans with OUD [25]. Given this physician shortage, surgeons are often faced with having to treat these patients without the assistance and expertise of an addiction specialist.

While 46% of surgeons reported feeling comfortable managing patients with OUD, this survey did not determine that perceived comfort translated into competence in the management of these patients. When asked how surgeons approached the care of these patients and harm reduction, almost half of respondents reported limiting the dose or duration of opioids in the postoperative time period. While this is an important aspect of harm reduction, this method may result in inadequate pain control. These patients have an increased tolerance to opioids and often require higher doses in the immediate postoperative period and surgeons may be reluctant to adequately treat with or prescribe the higher doses needed for fear of worsening their OUD. Interestingly, a study that looked at risks for relapse in patients with a history of OUD showed that uncontrolled or poorly controlled pain was the strongest risk factor for relapse as these patients may be driven to “self treat” their uncontrolled pain [26]. Given the results of this study, it is imperative that surgeons be knowledgeable and comfortable in adequately treating postoperative pain in this patient population to avoid inadvertently contributing to their risk of relapse.

When surgeons were asked if they had specialty or hospital specific guidelines regarding the treatment of opioid use disorder, 30% reported having guidelines available. However, when asked what methods they used to promote harm reduction and treat this population, only 2% reported using available guidelines. This suggests that while guidelines are available, only a small percent of surgeons consider these guidelines an important part of their practice. This question was asked as a free text question in which respondents were asked to list different methods they used. This may have resulted in respondents self-selecting which methods they found most useful in their daily practice. When asked if society sponsored guidelines or best practice statements would be helpful, 59% responded that they would like to have these guidelines available. The free response nature of this question may have also resulted in respondents only listing the primary method they use as opposed to multiple different strategies that they may actually be using. This discrepancy between reported use and surgeons requesting more guidelines suggests that the currently available guidelines are not widely used by surgeons in their daily practice. While many organizations have published guidelines regarding prevention of chronic opioid use and opioid use disorder, guidelines regarding patients with ongoing chronic opioid use or OUD are not as readily available.

Respondents were asked what they found to be the most common challenges faced when treating patients with OUD. Two thirds reported some form of lack of knowledge. This included unfamiliarity with medication dosing, concern about overprescribing or contributing to the OUD and concern about legal ramifications and liability of prescribing the higher doses of opioids needed to control patients’ pain. Many of the challenges described by surgeons related to a lack of knowledge included both a lack of formal training in how to appropriately treat pain in this population but also a concern that their efforts in providing appropriate analgesia could inadvertently worsen the patients’ OUD or risk recidivism in patients that are successfully being treated for OUD. Another third of responses included concerns about lack of resources. One of the most common concerns was a shortage of specialists available to assist with these patients’ care. This included addiction medicine specialists, psychiatrists and pain management physicians. Specialist availability was a challenge in both the inpatient setting as well as in the outpatient transitional phases of care. In some institutions, specialists are able to be consulted to assist in inpatient treatment but do not have the capacity to see additional patients in the outpatient setting and will not prescribe opioids on discharge. As this shortage of specialists is not likely to improve in the immediate future, actions need to be taken to assist surgeons in managing these patients in the postoperative phase if there is not a specialist available to take over their care. Several studies have used national databases to identify physician specialties that most often treat OUD and utilize MAT [27]. One article from Wen and colleagues found that internist and family medicine physicians prescribed 50% of medication assisted therapy. Perhaps there is an opportunity to involve primary care specialties more in the acute setting when caring for these patients. Another challenge related to physician shortage is the concern that surgeons will not be able to transition care to a specialist after the acute postoperative period and will be faced with long term prescribing of opioids for these patients. Interestingly, a study of Veteran’s Affairs patients undergoing general surgical procedures showed that chronic opioid users were able to return to their baseline opioid requirements in a similar time fashion as their opioid naïve counterparts [28]. This suggests that the concern about prescribing additional opioids to this patient population may not be as commonplace as previously thought.

Guidelines regarding the care of patients with OUD recommend the use of MAT when possible. This includes the use of methadone and/or buprenorphine. Multiple studies have shown this to be an effective way to treat both the existing OUD and can be adjusted to help control postoperative pain. While MAT is known to be beneficial to these patients, surgeons cite again both a lack of knowledge and formal training as well as a lack of resources. These guidelines may also vary from state to state. MAT is likely an underutilized resource when surgeons encounter these patients in the acute setting.

Lastly, surgeons were asked how they could be better supported when caring for patients with OUD. Most responses centered around increased availability of specialists that can be enlisted to help care for these patients. This question was initially asked in the setting of what psychiatrists could do to assist surgeons but certainly the responses can be extrapolated to whichever specialist primarily manages these patients at different institutions, whether that be psychiatry, addiction medicine, pain management, anesthesia or internal medicine. Surgeons also stated that it would be helpful to have a multidisciplinary team. In many institutions, pain management physicians, psychiatrists, pharmacists and social workers are all routinely employed to assist in the care of these patients but it is not implemented in a combined multidisciplinary approach. Interestingly, several respondents did not consider psychiatry as a specialty they would routinely involve in the treatment of OUD despite the specialty organization interest in caring for this population. There is an opportunity for increased awareness regarding which specialties are able to and willing to assist in the care of these patients. It would also be beneficial to have increased cooperation and collaboration between surgical and medical organizations to improve the treatment for these patients during the perioperative period.

As research efforts continue to focus on how best to treat patients’ perioperative pain, including those with OUD, the concept of “opiate free anesthesia” (OFA) is emerging as a method to decrease the use of opioids [29]. By using a multimodal nonopioid approach to anesthesia and perioperative analgesia, patients are able to avoid opioids altogether with either similar or improved outcomes compared to standard opioid analgesia [30–32]. A meta-analysis by D’Amico and colleagues examined the use of OFA in thoracic surgery and concluded that it was associated with lower rates of complications, lower pain scales and lower morphine consumption postoperatively [33]. While it remains to be seen how this could be utilized in patients with chronic opioid use and OUD, its effectiveness in decreasing perioperative opioid use may be an important method of harm reduction in the future.

While this survey highlighted important and common concerns that surgeons have regarding the challenges faced when caring for patients with OUD, there are several limitations to the study. While this survey had a similar response rate to many surveys conducted by the American College of Surgeons, the response rate still only represented a small fraction of its membership. There also may be a participant bias in those that chose to respond, with respondents being more likely to participate if this is a topic that they routinely face or have specific opinions regarding the treatment of OUD. It is possible that many surgeons do not face challenges when caring for patients with OUD and thus declined to take the survey.

Another limitation of the study was that the definitions of OUD and chronic opioid use were not expressly defined at the beginning of the survey. If respondents were unaware of the differences between these two populations, this could have resulted in confusion and inaccurate responses to the questions. While there are overlap in these populations regarding maintaining appropriate analgesia, there are many important differences between the two diagnoses.

Lastly, many of the answer choices in this survey were free text responses. While this was helpful in obtaining honest and practical answers from respondents, it may have limited responses or resulted in significant variability in the interpretation of the questions. For example, when surgeons were asked what methods they used when caring for patients with OUD, having a free response answer may have led respondents to simply choose their top two preferences or strategies, leaving out multiple strategies and tools that they may use. Future surveys may benefit from having multiple selection choices so that surgeons are able to include all possible strategies that they routinely implement.

This survey of the membership of the American College of Surgeons describes the challenges faced by surgeons when treating patients with OUD. Many barriers included a lack of training or lack of resources. There are opportunities for partnership between organizations to develop best practice guidelines to improve the care of these patients. Additional studies should be done to help identify what strategies are most effective in caring for these patients and how these can be implemented by surgeons in regions where specialists are not readily available. While surgeons have played an important role in the prevention of chronic opioid use and OUD through national education and advocacy efforts, it is important that we continue to be at the forefront in improving the care of patients already affected by opioid use disorder to ensure that when they are receiving the best care possible.

## Conclusion

Surgeons are frequently faced with caring for patients with opioid use disorder and providing appropriate analgesia. This study highlights the prevalence of this issue as well as surgeon discomfort in caring for these patients. The majority of surgeons rely on a consulting service to assist with the management of this population. Surgeons feel it would be beneficial to have formal best practice guidelines available to be able to provide these patients with the optimal care during the perioperative period.

## Data Availability

No datasets were generated or analysed during the current study.

## Abbreviations

OUD: Opioid Use Disorder
ACS: American College of Surgeons
MAT: Medication Assisted Therapy
PDMP: Prescription Drug Monitoring Program

## References

[1] Baltes A, Horton D, Malicki J, Trevino C, Agarwal S, Zarzuar B, Brown R (2024). Pain management in trauma: the need for trauma informed opioid prescribing guidelines. Trauma Surg Acute Care Open.

[2] Di Lena E, Barone N, Hopkins B, Do U, Kaneva P. Opioid prescribing practices in breast oncologic surgery; a retrospective cohort study. World J Surg. 2024; Online ahead of print 10.1002/wjs.12079 38312060

[3] Alzatari R, Huang L, Poulose B. The impact of opioid versus non-opioid analgesics on postoperative pain level, quality of life and outcomes in ventral hernia repair. Hernia. 2024; Online ahead of print 10.1007/s10029-024-02968-3 PMC11450054 38296871

[4] Stephenson K, Krinock D, Vasquez I, Shewmake C, Spray B, Ketha B, et al. Implementation of guidelines limiting postoperative opioid prescribing at a children’s hospital. J Patient Saf. 2024; Online ahead of print 10.1097/PTS.0000000000001209 38240645

[5] Zhang K, Blum K, Chu J, Zewdu A, Hanse S, Skoracki R, Janis J, Barker J (2023). A personalized opioid prescription model for predicting postoperative discharged opioid needs. Plast Reconstr Surg.

[6] Hilliard P, Lajee J, Moser S, Metz L, Mathis M, Goesling J, Cron D, Clauw D, Englesbe M, Abecasis G, Brummett C (2018). Prevalence of preoperative opioid use and characteristics associated with opioid use among patients presenting for surgery. JAMA Surg.

[7] Barot T, Zimmerman I, Daly J, Sachdeva A, Heneghan K (2019). American College of Surgeons: a systems level approach to the opioid epidemic and surgery.

[8] Lawal O, Gold J, Murthy A, Ruchi R, Bavry E, Hume A, Lewkowitz A, Brothers T, Wen X (2020). Rate and risk factors associated with prolonged opioid use after surgery: a systemic review and meta-analysis. JAMA Netw Open.

[9] Brummett C, Waljee J, Goesling J, Moser S, Lin P, Englesbe M, Bohnert A, Kheterpal S, Nallamothu B (2017). New persistent opioid use after minor and major surgical procedures in US adults. JAMA Surg..

[10] American Psychiatric Association (2013). Diagnostic and statistical manual of mental disorders.

[11] Levy B, Paulozzi L, Mack K, Jones C (2015). Trends in opioid analgesic-prescribing rates by specialty, US, 2007-2012. Am J Prev Med.

[12] Boltunova A, Bailey C, Weinberg R, Ma X, Thalappillil R, Tam C, White R (2020). Preoperative opioid use disorder is associated with poorer outcomes after coronary bypass and valve surgery: a multistate analysis, 2007-2014. J Cardiothorac Vasc Anesth.

[13] Lui B, Weinberg R, Milewski A, Alma X, Bustillo M, Mack P, White R (2021). Impact of preoperative opioid use disorder on outcomes following lumbar spine surgery. Clin Neurol Neurosurg.

[14] Dewan K, Dewan K, Idrees J, Navale S, Rosinski B, Svensson L, Gillinov A, Johnston D, Bakaeen F, Soltesz E (2019). Trends and outcomes of cardiovascular surgery in patients with opioid use disorders. JAMA Surg.

[15] Song Y, Tang R, Roses R, Fraker D, DeMatteo R, Kelz R, Karakousis G (2021). Opioid use disorder is associated with complications and increased length of stay after major abdominal surgery. Ann Surg.

[16] Veazie S, Mackey K, Bourne D, Peterson K (2019). Evidence brief: managing acute pain in patients with opioid use disorder on mediation-assisted treatment.

[17] Ayakta N, Sceats L, Merrel S, Kin C (2021). “It’s like learning by the seat of your pants”: surgeons lack formal training in opioid prescribing. J Surg Educ.

[18] Tiako M, Mszar R, Brooks C, Mahmood S, Mori M, Vallabhajosyula P, Geirsson A, Weimer M (2022). Cardiac surgeons’ practices and attitudes toward addiction care for patients with substance use disorders. Subst Abus.

[19] Han B, Compton W, Blanco C, Crane E, Lee J, Jones C (2017). Prescription opioid use, misuse and use disorders in US adults: 2015 National Survery on Drug Use and Health. Ann of Intern Med.

[20] Menendez M, Mellema J, Ring D (2015). Attitudes and self-reported practices of hand surgeons regarding prescription opioid use. Hand..

[21] Theodorou C, Jackson J, Rajaseka G, Nuno M, Yamashiro K, Farmer D, Hirose S, Brown E (2021). Impact of prescription drug monitoring program mandate on postoperative opioid prescriptions in children. Pediatr Surg Int.

[22] Wang T, Tong J, Hersh E, Chuang S, Panchal N (2021). Does prescription drug monitoring program usage affect opioid analgesic prescriptions by oral and maxillofacial surgeons after third molar surgery?. Oral Surg Oral Med Oral Pathol Oral Radiol.

[23] Gupta R, Boehmer S, Giampetro D, Gupta A, DeFlitch C (2021). Effect of a prescription drug monitoring program on emergency department opioid prescribing. West J Emerg Med.

[24] Dickerson D, Mariano E, Szokol J, Harned M, Clark R, Mueller J, et al. Multiorganizational consensus to define guiding principles for perioperative pain management in patients with chronic pain, preoperative opioid tolerance or substance use disorder. Reg Anesth Pain Med. 2023; online ahead of print. 10.1136/rapm-2023-104435 37185214

[25] Rosenblatt R, Andrilla C, Catlin M, Larson E (2015). Geographic and specialty distribution of US physicians trained to treat opioid use disorder. Ann Fam Med.

[26] Weiss R, Potter J, Fiellin D, Byrne M, Connery H, Dickinson W, Gardin J, Griffin M, Gourevitch M, Haller D, Hasson A, Huang Z, Jacobs P, Kosinski A, Lindblad R, Katz E, Provost S, Selzer J, Somoza E, Sonne S, Ling W (2011). Adjunctive counseling during brief and extended buprenorphine0naloxone treatment of prescription opioid dependence: a 2-phase randomized controlled trial. Arch Gen Psychiatry.

[27] Wen H, Borders T, Cummins J (2019). Trends in buprenorphine prescribing by physician specialty. Health Aff (Millwood).

[28] Bleicher J, Brooke B, Bayless K, Anderson Z, Beckstrom J, Zhang C, et al. Postoperative opioid prescribing, use and pain trends following general surgery procedures: a retrospective cohort study among veterans comparing non-opioid versus chronic opioid users. Reg Anesth Pain Med. 2022; Epub 10.1136/rapm-2021-103382 PMC10473845 35523480

[29] Cia P, Cannesson M, Bui C (2020). Opioid free anesthesia: feasible?. Curr Opin Anaesthesiol.

[30] Hao C, Xu H, Du J, Zhang T, Zhang X, Zhao Z, Luan H (2023). Impact of opioid-free aanesthesia on postoperative quality of recovery in patients after laparoscopic cholecystectomy – a randomized controlled trial. Drug Des Devel Ther.

[31] Burkhard J, Jardot F, Furrer M, Engel D, Beilstein C, Wuethrich P (2023). Opioid-free anesthesia for open radical cystectomy is feasible and accelerates return of bowel function: a matched cohort study. J Clin Med.

[32] Chen L, He W, Liu X, Lv F, Li Y (2023). Application of opioid-free general anesthesia for gynecological laparoscopic surgery under ERAS protocol: a non-inferiority randomized controlled trial. BMC Anesth.

[33] D’Amico F, Barucco G, Licheri M, Valsecchi G, Zaraca L, Mucchetti M, Zangrillo A, Monaco F (2022). Opioid free anesthesia in thoracic surgery: a systematic review and meta analysis. J Clin Med.

